# *Flame Retardancy Index* (*FRI*) for Polymer Materials Ranking

**DOI:** 10.3390/polym15112422

**Published:** 2023-05-23

**Authors:** Henri Vahabi, Elnaz Movahedifar, Baljinder K. Kandola, Mohammad Reza Saeb

**Affiliations:** 1 Université de Lorraine, CentraleSupélec, LMOPS, F-57000 Metz, France; el.movahedifar@gmail.com; 2 Institute for Materials Research and Innovation, University of Bolton, Bolton BL3 5AB, UK; b.kandola@bolton.ac.uk; 3 Department of Polymer Technology, Faculty of Chemistry, Gdańsk University of Technology, G. Narutowicza 11/12, 80-233 Gdańsk, Poland

**Keywords:** *FRI*, flame retardancy, polymer composites, cone calorimetry, micro calorimetry, PCFC, fire analysis

## Abstract

In 2019, we introduced *Flame Retardancy Index* (*FRI*) as a universal dimensionless index for the classification of flame-retardant polymer materials (*Polymers,* 2019, *11*(3), 407). *FRI* simply takes the peak of Heat Release Rate (pHRR), Total Heat Release (THR), and Time-To-Ignition (*t*_i_) from cone calorimetry data and quantifies the flame retardancy performance of polymer composites with respect to the blank polymer (the reference sample) on a logarithmic scale, as of *Poor* (*FRI* ˂ 10^0^), *Good* (10^0^ ≤ *FRI* ˂ 10^1^), or *Excellent* (*FRI* ≥ 10^1^). Although initially applied to categorize thermoplastic composites, the versatility of *FRI* was later verified upon analyzing several sets of data collected from investigations/reports on thermoset composites. Over four years from the time *FRI* was introduced, we have adequate proof of *FRI* reliability for polymer materials ranking in terms of flame retardancy performance. Since the mission of *FRI* was to roughly classify flame-retardant polymer materials, its simplicity of usage and fast performance quantification were highly valued. Herein, we answered the question “does inclusion of additional cone calorimetry parameters, e.g., the time to pHRR (*t*_p_), affect the predictability of *FRI*?”. In this regard, we defined new variants to evaluate classification capability and variation interval of *FRI*. We also defined the *Flammability Index* (*FI*) based on Pyrolysis Combustion Flow Calorimetry (PCFC) data to invite specialists for analysis of the relationship between the *FRI* and *FI*, which may deepen our understanding of the flame retardancy mechanisms of the condensed and gas phases.

## 1. Background

To pursue the effectiveness of flame retardants (FRs) incorporated into polymer materials, one essentially needs to monitor and understand the mechanism of action of FRs in the gas and condensed phases, individually or simultaneously [1]. Cone calorimetry is a well-known bench-scale testing method for the quantification of flame retardancy performance of polymers, which offers useful information about FRs’ action, particularly when it comes with barrier mechanism of the condensed phase. Since highly flame-retardant polymers generally benefit from the synergistic effects of two or more types of FRs, additional morphological and/or elemental composition analyses on char residue are required to explain the action of FRs [2]. Cone calorimetry analysis makes it possible to explore fire behavior/scenarios in terms of the heat release rate (HRR) as a function of time [3]. However, judgments about the superiority of a given FR added to a certain polymer over other FRs of the same or different categories are mostly fuzzy, arising from the incorrect interpretation of the results. In many cases, researchers discuss about the performance merely based on the reduction in the peak of HRR (pHRR) extracted from cone calorimetry curve, which leads to serious confusions as to whether or not the mechanism of flame retardancy was understood [1]. We comprehended that presumption about the performance of FRs in complex polymer composites containing two or more FRs necessitates using a universal index in order to categorize the flame-retardant polymers in view of FRs effectiveness.

In 1997, *Richard E. Lyon* et al. [4] defined Flame Spread Index (FSI) and Flame Propagation Index (FPI) with the involvement of the peak of HRR (pHRR) and Time-To-Ignition (*t*_i_) as explanatory parameters of typical cone calorimetry curves. These indices were incredible measures for the analysis of the flame retardancy of polymer materials as a qualitative measure of the speed of the flame front of a composite polymer. Although several other indices have been proposed and applied for the analysis of flame retardant polymers based on cone calorimetry data, there was a need for a universal index to simply categorize polymer composites in terms of a flame retardancy index. In 2019, inspired by the aforementioned pioneering work, we conceptualized the *Flame Retardancy Index* (*FRI*) as a self-explanatory index to categorize flame-retardant polymer materials by using Equation (1) [5]. The *FRI* was dimensionless and additionally included the Total Heat Release (THR), to quantify the flame retardancy performance of polymer composites with respect to the blank (reference) polymer.

(1) $$FRI=\frac{{\left[\left(\frac{pHRR}{t_{i}}\right)\times THR\right]}_{Neat\;Polymer}}{{\left[\left(\frac{pHRR}{t_{i}}\right)\times THR\right]}_{Composite}}$$

**Figure 1:**
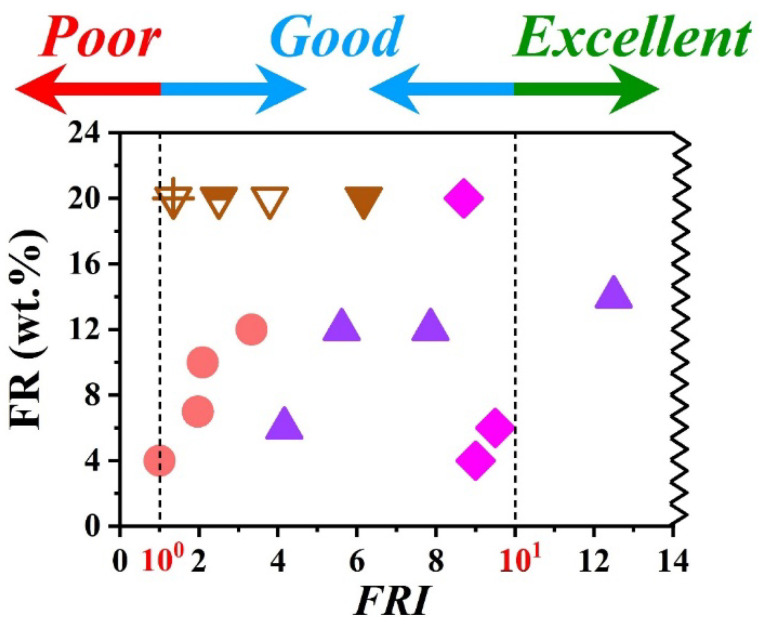
Alteration of flame retardancy performance of RFs applying *FRI* on a logarithmic scale. A series of hypothetical cone calorimetry data are assigned to polymer composites virtually to explain and mimic the of behavior of flame-retardant polymer materials, where *FRI* fluctuated upon variation of weight percent (wt.%) of FRs.

Figure 1 attempts to conceptualize the applicability of the *FRI* on a logarithmic scale, with *Poor* (*FRI* ˂ 10^0^), *Good* (10^0^ ≤ *FRI* ˂ 10^1^), and *Excellent* (*FRI* ≥ 10^1^) performance regions. Filled symbols in Figure 1 are representative of systems in which a given type of FR is used, but the addition of FRs (depending on the chemistry and amount) would change the flame retardancy performance. For instance, circles suggest the positive effect of the addition of the used FR, but the category of polymers with *Good* performance label do not change upon increasing FR content from 4 to 12 wt.%. For filled triangles, however, the classification of flame retardancy varies from *Good* to *Excellent* with almost identical FR wt.% variation interval. Above 12 wt.% loading, which seems to be a threshold of FR loading, a phenomenon might have happened, e.g., intumescent action or substantial charring effect. Rhomboids reveal a completely different behavior for that abstract polymer composite, such that *FRI* remains unchanged in the *Good* zone regardless of FR loading increase from 4 to 20 wt.%. The overturned triangles represent polymer materials in which FR content is fixed, but the type, combination, or chemical modification of FR would be the reason for higher efficiency, even though the category remains *Good*. These assumed cases would explain the worth of the *FRI* for polymer materials ranking. In practice, we examined the versatility of *FRI* in classifying flame retardant thermoplastic and thermoset polymer composites, along with the applicability of the proposed logarithmic scale for the reliability of mapping *Poor*-*Good*-*Excellent* territorial zones and borderlines in between zones. We extensively studied and classified flame-retardant polypropylene (PP) [6] and epoxy (EP) [7] polymer composites, as typical of the thermoplastic and thermoset polymer composites, respectively. Inspired by such important works, some research groups attempted to graphically correlate the *FRI* variation with other flame tests, e.g., UL-94 and Limited Oxygen Index (LOI) [8]. More examples of the successful implementation of the *FRI* in the quantification of the flame retardancy performance of polymers have been reported by other research groups, which are available for inquisitive readers [9,10,11]. In a very recent paper, Artificial Intelligence (AI) based modeling witnessed the reliability of the *FRI* for designing flame retardant polymers [12], which has been initially emphasized by our group [13].

## 2. Conceptualization 

**Figure 2:**
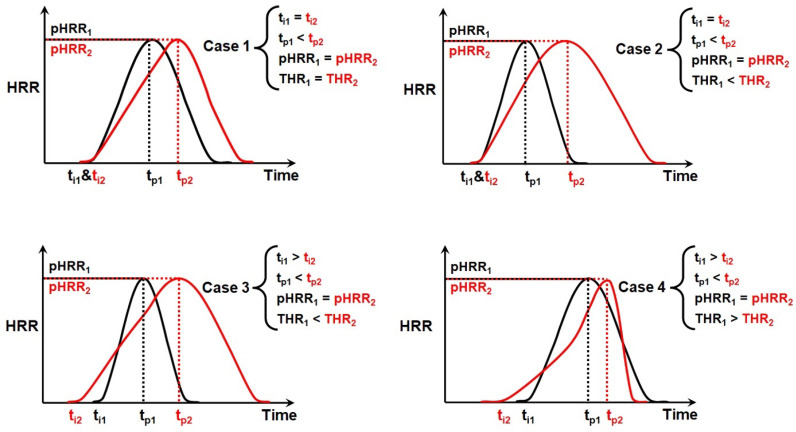
Some abstract fire scenarios (black and red in each scenario are representative of the typical behavior of samples) featuring cone calorimetry curves with different shapes, where *t_p_* and *t*_i_ are determinative parameters. The parameter pHRR is kept fixed in all abstract scenarios for simple comparison of cases in terms of THR and *t*_i_.

Although *FRI* cannot exclusively play a decisive role in the selection of FR for polymers, we have a strong proof of *FRIs* fitness for ranking semi-qualitative flame retardant polymer materials. Complications may arise from complex fire scenarios observed in cone calorimetry curves. The parameters of cone calorimetry may be identical or different for a given polymer material containing various types and amounts of FRs. Figure 2 compares four abstract cases in which the times to pHRR (*t*_p_) and *t_i_* are variable for a system taking pHRR value unchanged. Reduction in the value of pHRR has been mostly taken by the researchers as the only measure of the performance of FRs. Keeping the pHRR constant in Figure 2 may in a better way underline the importance of variation of *t_i_* and THR values. 

The first objective of this short communication is to introduce the idea of revisiting the *FRI* ranking potential by individual/combined inclusion of *t_i_* or/and *t_p_*. In other words, we are inquisitive to know whether or not the category of flame retardancy determined by *FRI* change by the individual or combined inclusion of *t_p_* and/or *t_i_* in the *FRI* formula. Three possible variants of *FRI* are defined as per Equations (2)–(4). Although the parameters in Equation (2) are not nominally the same as of those in Equation (1), both equations are conceptually identical. Correspondingly, possible correlations between the *FRI* values are visualized as a function of FRs weight percent (wt.%) for a number of flame retardant polymer materials based on the availability and authenticity of data collected from the literature (Table 1).

(2) $$FRI(t_{i})=\frac{{\left[\left(\frac{pHRR}{t_{i}}\right)\times THR\right]}_{Neat\;Polymer}}{{\left[\left(\frac{pHRR}{t_{i}}\right)\times THR\right]}_{Composite}}$$

(3) $$FRI(t_{p})=\frac{{\left[\left(\frac{pHRR}{t_{p}}\right)\times THR\right]}_{Neat\;Polymer}}{{\left[\left(\frac{pHRR}{t_{p}}\right)\times THR\right]}_{Composite}}$$

(4) $$FRI(t_{i}\&t_{p})=\frac{{\left[\left(\frac{pHRR}{t_{p}}\right)\times \left(\frac{THR}{t_{i}}\right)\right]}_{Neat\;Polymer}}{{\left[\left(\frac{pHRR}{t_{p}}\right)\times \left(\frac{THR}{t_{i}}\right)\right]}_{Composite}}.$$

PCFC is a well-known test for the analysis of flammability of polymer materials [26]. The second objective of this work was to introduce the concept of the *Flammability Index* (*FI*) based on PCFC data, as per Equation (5).

(5) $$FI=\frac{{\left[\left(\frac{pHRR}{T_{p}}\right)\times THR\right]}_{Neat\;Polymer}}{{\left[\left(\frac{pHRR}{T_{p}}\right)\times THR\right]}_{Composite}}$$

The correlation between *FRI* and *FI* may deepen our understanding of the correlation between the flame retardancy mechanisms in the condensed and gas phases. The extensions given to the *FRI* concept or possible variants of *FRI* as well as newly introduced *FI* are correlated based on very limited statistics. This necessitates the collection of a larger pool of data in the future. Thus, we strongly advise that any blind or exaggerative generalization of the outcomes of this survey in terms of *FRI* and *FI* for demonstrating fire behavior of polymer materials should be censoriously avoided.

## 3. Visualization

**Figure 3:**
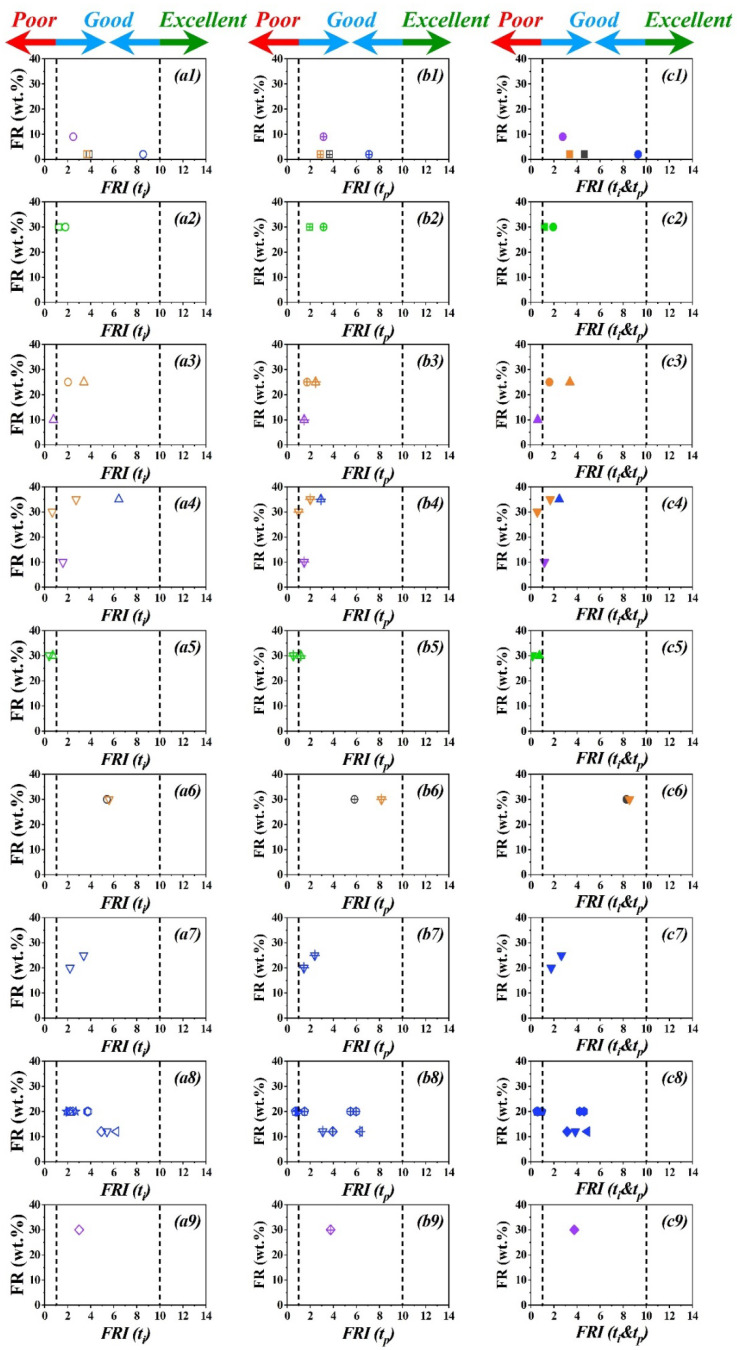
Graphical view of variation of *FRI*, i.e., *FRI* (*t_i_*) (‘**a**’ series), *FRI* (*t_p_*) (‘**b**’ series), *FRI* (*t_i_*&*t_p_*) (‘**c**’ series) per polymer as a function of FR content (wt.%) based on data summarized in Table 1. Columns 1, 2 and 3 are named **a1**–**a9**, **b1**–**b9**, and **c1**–**c9**, in the same order. At each row we have a polymer, which is named in correspondence with the symbols and tables. Symbols of the same color and shape are used as representatives of the type of FR and the amount of FR. Plots are shown per nine polymer type available in the literature; *FRI* (*t_i_*) symbols are 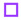 EP/DP-27.3 [14], Si-EP/DP-27.3 [14], 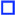 EP/DP-27.3/Mel-9.1 [14], Si-EP/DP-27.3/Mel-9.1 [14], 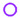 EP/BPA-BPP-9 [15], 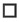 EP/GN-2 [16], 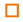 EP/Ni–Fe LDH-2 [16], 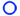 EP/NiFe-LDH&GN-2 [16], 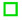 ABS/LIG-30 [17], 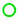 ABS/P-LIG-30 [17], 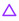 EVA/APP-10 [18], 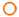 EVA/ATH-25 [19], 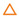 EVA/Boehm-25 [19], 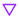 Cop-PA66&PA6/SiDOPO-10 [20], 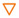 PA66/GF-35 [21], PA12/GF-30 [21], PEEK/GF-30 [23], 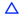 PA66/GF&RP-35 [21], 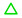 PBS/Cellulose-30 [22], 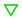 PBS/Bamboo-30 [22], 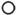 PEEK/CF-30 [23], 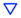 PP/BrFR&Sb_2_O_3_-20 [24], PP/BrFR&Sb_2_O_3_-25 [24], PS/BrFR&Sb_2_O_3_-12 [24], 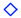 PS/BrFR&Sb_2_O_3_-10/C15A-2 [24], 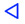 PS/BrFR&Sb_2_O_3_-10/MWCNT-2 [24], 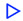 PS/APP&TPER-20 [25], 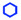 PS/APP&TPER-19/C15A-1 [25], PS/APP&TPER-18//C15A-2 [25], 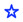 PS/APP&TPER-19/MWCNT-1 [25], PS/APP&TPER-18/MWCNT-2 [25], 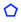 PS/APP&TPER-19/Fe_2_O_3_-1 [25], PS/APP&TPER-18/Fe_2_O_3_-2 [25], 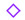 TPES/AlPi-30 [21]; *FRI* (*t_p_*) symbols are 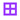 EP/DP-27.3 [14], Si-EP/DP-27.3 [14], 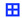 EP/DP-27.3/Mel-9.1 [14], Si-EP/DP-27.3/Mel-9.1 [14], 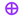 EP/BPA-BPP-9 [15], 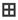 EP/GN-2 [16], 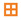 EP/Ni–Fe LDH-2 [16], 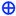 EP/NiFe-LDH&GN-2 [16], 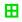 ABS/LIG-30 [17], 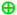 ABS/P-LIG-30 [17], 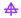 EVA/APP-10 [18], 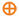 EVA/ATH-25 [19], 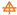 EVA/Boehm-25 [19], 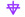 Cop-PA66&PA6/SiDOPO-10 [20], 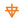 PA66/GF-35 [21], PA12/GF-30 [21], PEEK/GF-30 [23], 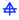 PA66/GF&RP-35 [21], 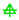 PBS/Cellulose-30 [22], 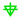 PBS/Bamboo-30 [22], 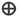 PEEK/CF-30 [23], 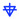 PP/BrFR&Sb_2_O_3_-20 [24], PP/BrFR&Sb_2_O_3_-25 [24], PS/BrFR&Sb_2_O_3_-12 [24], 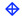 PS/BrFR&Sb_2_O_3_-10/C15A-2 [24], 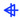 PS/BrFR&Sb_2_O_3_-10/MWCNT-2 [24], 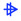 PS/APP&TPER-20 [25], 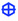 PS/APP&TPER-19/C15A-1 [25], PS/APP&TPER-18//C15A-2 [25], 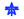 PS/APP&TPER-19/MWCNT-1 [25], PS/APP&TPER-18/MWCNT-2 [25], 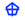 PS/APP&TPER-19/Fe_2_O_3_-1 [25], PS/APP&TPER-18/Fe_2_O_3_-2 [25], 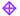 TPES/AlPi-30 [21]; and *FRI(t_i_&t_p_*) symbols are 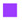 EP/DP-27.3 [14], Si-EP/DP-27.3 [14], 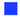 EP/DP-27.3/Mel-9.1 [14], Si-EP/DP-27.3/Mel-9.1 [14], 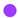 EP/BPA-BPP-9 [15], 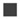 EP/GN-2 [16], 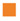 EP/Ni–Fe LDH-2 [16], 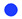 EP/NiFe-LDH&GN-2 [16], 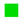 ABS/LIG-30 [17], 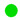 ABS/P-LIG-30 [17], 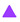 EVA/APP-10 [18], 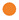 EVA/ATH-25 [19], 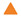 EVA/Boehm-25 [19], 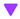 Cop-PA66&PA6/SiDOPO-10 [20], 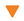 PA66/GF-35 [21], PA12/GF-30 [21], PEEK/GF-30 [23], 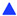 PA66/GF&RP-35 [21], 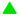 PBS/Cellulose-30 [22], 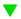 PBS/Bamboo-30 [22], 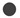 PEEK/CF-30 [23], 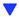 PP/BrFR&Sb_2_O_3_-20 [24], PP/BrFR&Sb_2_O_3_-25 [24], PS/BrFR&Sb_2_O_3_-12 [24], 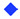 PS/BrFR&Sb_2_O_3_-10/C15A-2 [24], 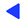 PS/BrFR&Sb_2_O_3_-10/MWCNT-2 [24], 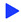 PS/APP&TPER-20 [25], 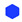 PS/APP&TPER-19/C15A-1 [25], PS/APP&TPER-18//C15A-2 [25], 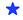 PS/APP&TPER-19/MWCNT-1 [25], PS/APP&TPER-18/MWCNT-2 [25], 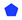 PS/APP&TPER-19/Fe_2_O_3_-1 [25], PS/APP&TPER-18/Fe_2_O_3_-2 [25], 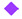 TPES/AlPi-30 [21].

Figure 3 displays the variation of possible *FRI* variants, i.e., *FRI* (*t_i_*), *FRI* (*t_p_*), and *FRI* (*t_i_*&*t_p_*) as a function of FR (wt.%). Each line is representative of a given polymer. It is crucial to emphasize that the performance category (*Poor*, *Good*, or *Excellent*) is not changed by the inclusion of *t_i_* and/or *t_p_* in the *FRI* formula, whatever the mechanism of action of FR. For example, in the case of ATH (symbols 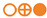, Figure 3(a3,b3,c3)), with the main action of the dilution of gas phase by releasing water, the *FRI* values are quite similar and the classification remains unchanged. Another example is brominated FRs (symbols 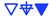, Figure 3(a7,b7,c7)), acting in the gas phase through free radical capture; again, the *FRI* values are quite similar. Aluminum diethyl phosphinate (AlPi) (symbols 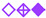, Figure 3(a9,b9,c9)) can also be considered as typical phosphorus FRs acting essentially in the gas phase. However, the *FRI* values should be sensitive to systems in which the main flame retardancy action is contributed by the condensed phase. Even a synergistic effect could be detected by a combination of several FRs. The difference in the *FRI* values has more pronounced in the case of the combination/synergism between ammonium polyphosphate (APP), tripentaerythritol (TPER), and multiwall carbon nanotubes (MWCNT) acting in the condensed phase. This could be explained by the increase in the value of *t_p_* resulting from the barrier effect and the resilience of the material to liberate the combustion gases followed by apparition of the peak. Noteworthily, classification in terms of “*Poor*”, “*Good*”, or “*Excellent*” has remained unchanged, except for one system (PA/GF/RP). For polyamide containing glass fiber and red phosphorus (symbols 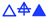, Figure 3(a4,b4,c4)), the value of the *FRI* (*t_i_*) is 6.44, while for *FRI* (*t_p_*) it is 2.92. This difference can be explained by the presence of glass fiber and the special case of the “candle wick” effect during combustion with a shorter time to reach pHRR [27]. First, it can be concluded that the *FRI* is able to classify all types of systems whatever the action of flame retardant in the condensed or gas phase, and also regardless of the type of polymer. Second, it is difficult to reach an *Excellent* level of flame retardancy, such that only a hybrid FR system representing different actions of flame retardancy may be needed to reach *FRI* values above 10.

**Figure 4:**
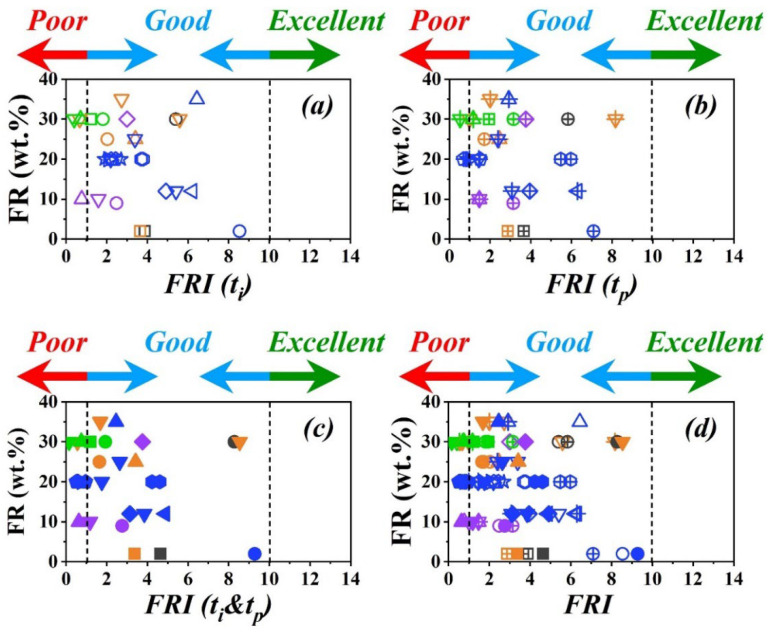
Graphical view of variation of *FRI*, i.e., *FRI* (*t_i_*) (**a**), *FRI* (*t_p_*) (**b**), *FRI* (*t_i_*&*t_p_*) (**c**), and four-in-one (**d**), regardless of polymer type, as a function of FR content (wt.%) based on data in Table 1. Symbols of the same color and shape are used as representatives of the type of FR, and amount of FR.; *FRI* (*t_i_*) symbols are 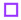 EP/DP-27.3 [14], Si-EP/DP-27.3 [14], 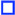 EP/DP-27.3/Mel-9.1 [14], Si-EP/DP-27.3/Mel-9.1 [14], 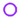 EP/BPA-BPP-9 [15], 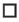 EP/GN-2 [16], 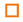 EP/Ni–Fe LDH-2 [16], 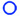 EP/NiFe-LDH&GN-2 [16], 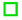 ABS/LIG-30 [17], 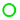 ABS/P-LIG-30 [17], 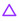 EVA/APP-10 [18], 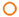 EVA/ATH-25 [19], 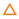 EVA/Boehm-25 [19], 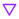 Cop-PA66&PA6/SiDOPO-10 [20], 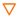 PA66/GF-35 [21], PA12/GF-30 [21], PEEK/GF-30 [23], 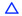 PA66/GF&RP-35 [21], 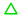 PBS/Cellulose-30 [22], 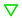 PBS/Bamboo-30 [22], 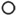 PEEK/CF-30 [23], 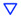 PP/BrFR&Sb_2_O_3_-20 [24], PP/BrFR&Sb_2_O_3_-25 [24], PS/BrFR&Sb_2_O_3_-12 [24], 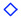 PS/BrFR&Sb_2_O_3_-10/C15A-2 [24], 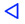 PS/BrFR&Sb_2_O_3_-10/MWCNT-2 [24], 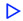 PS/APP&TPER-20 [25], 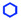 PS/APP&TPER-19/C15A-1 [25], PS/APP&TPER-18//C15A-2 [25], 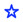 PS/APP&TPER-19/MWCNT-1 [25], PS/APP&TPER-18/MWCNT-2 [25], 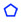 PS/APP&TPER-19/Fe_2_O_3_-1 [25], PS/APP&TPER-18/Fe_2_O_3_-2 [25], 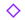 TPES/AlPi-30 [21]; *FRI* (*t_p_*) symbols are 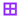 EP/DP-27.3 [14], Si-EP/DP-27.3 [14], 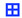 EP/DP-27.3/Mel-9.1 [14], Si-EP/DP-27.3/Mel-9.1 [14], 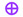 EP/BPA-BPP-9 [15], 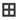 EP/GN-2 [16], 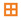 EP/Ni–Fe LDH-2 [16], 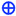 EP/NiFe-LDH&GN-2 [16], 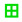 ABS/LIG-30 [17], 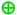 ABS/P-LIG-30 [17], 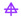 EVA/APP-10 [18], 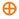 EVA/ATH-25 [19], 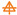 EVA/Boehm-25 [19], 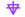 Cop-PA66&PA6/SiDOPO-10 [20], 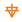 PA66/GF-35 [21], PA12/GF-30 [21], PEEK/GF-30 [23], 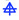 PA66/GF&RP-35 [21], 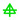 PBS/Cellulose-30 [22], 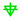 PBS/Bamboo-30 [22], 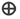 PEEK/CF-30 [23], 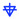 PP/BrFR&Sb_2_O_3_-20 [24], PP/BrFR&Sb_2_O_3_-25 [24], PS/BrFR&Sb_2_O_3_-12 [24], 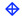 PS/BrFR&Sb_2_O_3_-10/C15A-2 [24], 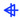 PS/BrFR&Sb_2_O_3_-10/MWCNT-2 [24], 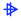 PS/APP&TPER-20 [25], 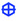 PS/APP&TPER-19/C15A-1 [25], PS/APP&TPER-18//C15A-2 [25], 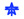 PS/APP&TPER-19/MWCNT-1 [25], PS/APP&TPER-18/MWCNT-2 [25], 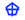 PS/APP&TPER-19/Fe_2_O_3_-1 [25], PS/APP&TPER-18/Fe_2_O_3_-2 [25], 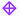 TPES/AlPi-30 [21]; and *FRI(t_i_&t_p_*) symbols are 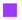 EP/DP-27.3 [14], Si-EP/DP-27.3 [14], 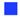 EP/DP-27.3/Mel-9.1 [14], Si-EP/DP-27.3/Mel-9.1 [14], 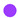 EP/BPA-BPP-9 [15], 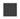 EP/GN-2 [16], 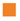 EP/Ni–Fe LDH-2 [16], 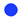 EP/NiFe-LDH&GN-2 [16], 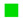 ABS/LIG-30 [17], 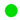 ABS/P-LIG-30 [17], 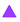 EVA/APP-10 [18], 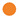 EVA/ATH-25 [19], 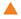 EVA/Boehm-25 [19], 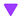 Cop-PA66&PA6/SiDOPO-10 [20], 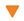 PA66/GF-35 [21], PA12/GF-30 [21], PEEK/GF-30 [23], 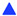 PA66/GF&RP-35 [21], 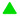 PBS/Cellulose-30 [22], 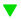 PBS/Bamboo-30 [22], 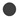 PEEK/CF-30 [23], 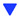 PP/BrFR&Sb_2_O_3_-20 [24], PP/BrFR&Sb_2_O_3_-25 [24], PS/BrFR&Sb_2_O_3_-12 [24], 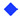 PS/BrFR&Sb_2_O_3_-10/C15A-2 [24], 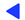 PS/BrFR&Sb_2_O_3_-10/MWCNT-2 [24], 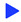 PS/APP&TPER-20 [25], 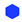 PS/APP&TPER-19/C15A-1 [25], PS/APP&TPER-18//C15A-2 [25], 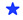 PS/APP&TPER-19/MWCNT-1 [25], PS/APP&TPER-18/MWCNT-2 [25], 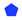 PS/APP&TPER-19/Fe_2_O_3_-1 [25], PS/APP&TPER-18/Fe_2_O_3_-2 [25], 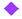 TPES/AlPi-30 [21].

Figure 4 shows the variation of *FRI* (*t_i_*), *FRI* (*t_p_*), and *FRI* (*t_i_*&*t_p_*) as a function of FR (wt.%) regardless of the polymer type. In general, the efficiency of each FR system at a given loading percentage remains almost the same whatever the type of polymer. For example, biobased FRs, such as lignin cellulose and bamboo (symbols 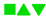, Figure 4c), slightly improve the flame retardancy even at a high loading percentage of 30 wt.%. In the case of phosphorus FR with well-known action in the condensed phase, however, FR percentage is a flammability-determining factor. Obviously, it is possible to obtain a high flame retardancy (symbol 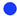, Figure 4c) even at a very low loading percentage., e.g., by combining LDH with graphene in EP. Based on the statistics used in this survey, the use of *FRI* (*t_i_*), *FRI* (*t_p_*), or *FRI* (*t_i_*&*t_p_*) does not change the category of flame retardancy performance. However, a larger pool of data would be required for analyzing the performance of FRs depending on the *FRI* formula. The pHRR will be weighted on the time at which the maximum heat release rate is recorded, while the THR will be weighted on the time at which the flaming phenomenon starts, which directly affects the area under the HRR curve. Thus, the FSI and FPI measures already defined by *Lyon* and coworkers [4] would change significantly when a flame-sensitive polymers is under measurement. Correspondingly, the *FRI* (*t_i_*), *FRI* (*t_p_*), or *FRI* (*t_i_*&*t_p_*) change in a manner that the classification might severely depend on the *FRI* formula.

The effects of synergistic combinations of FRs on the flame retardancy performance of polymer materials have been quite repeatedly claimed by researchers. Only a few examples can be found in the literature, which are summarized in Table 2.

**Figure 5:**
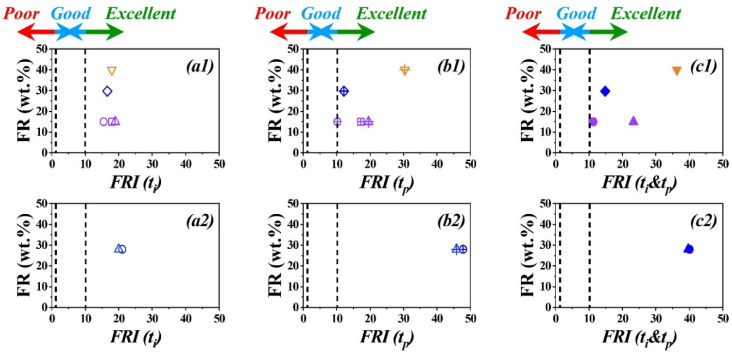
Graphical view of “*Excellent*” class of EP (**top plots**) and PP (**bottom plots**) flame-retardant polymers, as two typical polymers among thermosets and thermoplastics families, respectively, based on data in Table 2. The variants of *FRI*, i.e., *FRI* (*t_i_*) (**a1**,**a2**), *FRI* (*t_p_*) (**b1**,**b2**), and *FRI* (*t_i_*&*t_p_*) (**c1**,**c2**) are plotted for each polymer. For highly efficient flame retardant polymers comprising hybridized FRs, a big shift is observed when using *FRI* (*t_p_*) and *FRI* (*t_i_*&*t_p_*) with respect to *FRI* (*t_i_*) at a given FR content (wt.%). Symbols are conceptually similar, where 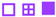 EP/PAz-APP-15 [28], 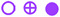 EP/APP-15 [29], 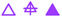 EP/GMA-APP-15 [29], 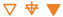 EP/BO-40 [30], 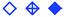 EP/Mel-APP/Talc-29.7 [31], 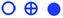 PP/IFR/OTAB-MMT-28 [32], 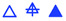 PP/IFR/A-POSS-28 [32].

Analyses based on *FRI* (*t_i_*), *FRI* (*t_p_*), and *FRI* (*t_i_*&*t_p_*) as a function of FR content (wt.%) for PP and EP systems are patterned in Figure 5. First, it was proved that the high *FRI* values leading to “*Excellent*” flame retardancy performance are possible just by a combination of two, or the hybridization of more than two FRs with different actions. For instance, phosphorus- and nitrogen-based FRs combined with inorganic fillers guaranteed a synergistic effect [32]. Second, the *FRI* values are not significantly changed in the case of *FRI* (*t_i_*), *FRI* (*t_p_*), and *FRI* (*t_i_*&*t_p_*), such that the “*Excellent*” class of flame retardancy performance remained unchanged. According to Table 2, *FRI* (*t_i_*) values may increase from almost 20 to 40–50, as calculated by *FRI* (*t_p_*) and *FRI* (*t_i_*&*t_p_*). This large difference is indicative of the sensitivity of HRR to a system containing two or more FRs in which a complicated fire scenario depends on the action of FRs in the gas and/or condensed phases. This is a proof that the variants of *FRI* may unveil the superiority of complex FR systems by a large shift in *FRI* (*t_p_*) or *FRI* (*t_i_*&*t_p_*) with respect to the *FRI* (*t_i_*), characteristic of the role of *t_p_* for systems with the “*Excellent*” performance. Therefore, the fire scenario largely affects the calculation and interpretation of *FRI* results. Overall, *FRI* remains reliable for the classification of flame retardant polymer materials, even in the “*Excellent*” zone.

## 4. Discussion

**Figure 6:**
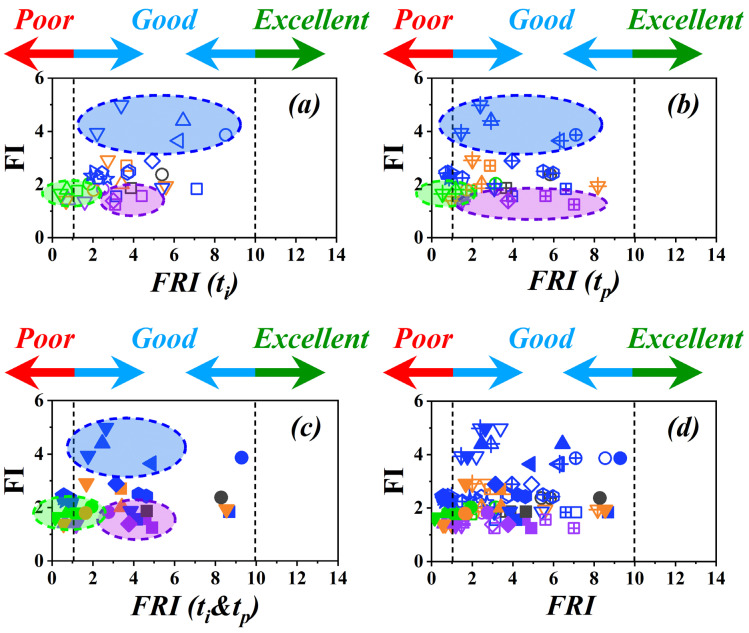
Graphical view of variation of *FI* versus different variants of *FRI*, i.e., *FRI* (*t_i_*) (**a**), *FRI* (*t_p_*) (**b**), *FRI* (*t_i_*&*t_p_*) (**c**), and four-in-one (**d**), plotted based on data in Table 1. Symbols of the same color and shape are used as representatives of the type of FR, but the amount of FR remains unseen; *FRI* (*t_i_*) symbols are 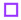 EP/DP-27.3 [14], Si-EP/DP-27.3 [14], 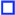 EP/DP-27.3/Mel-9.1 [14], Si-EP/DP-27.3/Mel-9.1 [14], 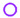 EP/BPA-BPP-9 [15], 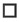 EP/GN-2 [16], 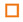 EP/Ni–Fe LDH-2 [16], 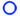 EP/NiFe-LDH&GN-2 [16], 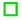 ABS/LIG-30 [17], 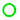 ABS/P-LIG-30 [17], 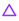 EVA/APP-10 [18], 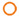 EVA/ATH-25 [19], 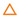 EVA/Boehm-25 [19], 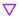 Cop-PA66&PA6/SiDOPO-10 [20], 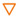 PA66/GF-35 [21], PA12/GF-30 [21], PEEK/GF-30 [23], 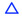 PA66/GF&RP-35 [21], 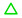 PBS/Cellulose-30 [22], 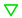 PBS/Bamboo-30 [22], 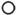 PEEK/CF-30 [23], 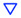 PP/BrFR&Sb_2_O_3_-20 [24], PP/BrFR&Sb_2_O_3_-25 [24], PS/BrFR&Sb_2_O_3_-12 [24], 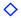 PS/BrFR&Sb_2_O_3_-10/C15A-2 [24], 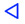 PS/BrFR&Sb_2_O_3_-10/MWCNT-2 [24], 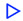 PS/APP&TPER-20 [25], 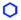 PS/APP&TPER-19/C15A-1 [25], PS/APP&TPER-18//C15A-2 [25], 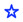 PS/APP&TPER-19/MWCNT-1 [25], PS/APP&TPER-18/MWCNT-2 [25], 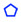 PS/APP&TPER-19/Fe_2_O_3_-1 [25], PS/APP&TPER-18/Fe_2_O_3_-2 [25], 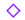 TPES/AlPi-30 [21]; *FRI* (*t_p_*) symbols are 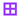 EP/DP-27.3 [14], Si-EP/DP-27.3 [14], 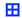 EP/DP-27.3/Mel-9.1 [14], Si-EP/DP-27.3/Mel-9.1 [14], 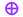 EP/BPA-BPP-9 [15], 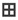 EP/GN-2 [16], 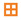 EP/Ni–Fe LDH-2 [16], 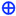 EP/NiFe-LDH&GN-2 [16], 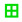 ABS/LIG-30 [17], 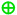 ABS/P-LIG-30 [17], 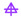 EVA/APP-10 [18], 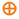 EVA/ATH-25 [19], 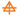 EVA/Boehm-25 [19], 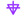 Cop-PA66&PA6/SiDOPO-10 [20], 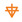 PA66/GF-35 [21], PA12/GF-30 [21], PEEK/GF-30 [23], 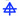 PA66/GF&RP-35 [21], 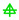 PBS/Cellulose-30 [22], 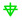 PBS/Bamboo-30 [22], 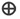 PEEK/CF-30 [23], 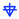 PP/BrFR&Sb_2_O_3_-20 [24], PP/BrFR&Sb_2_O_3_-25 [24], PS/BrFR&Sb_2_O_3_-12 [24], 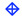 PS/BrFR&Sb_2_O_3_-10/C15A-2 [24], 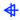 PS/BrFR&Sb_2_O_3_-10/MWCNT-2 [24], 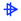 PS/APP&TPER-20 [25], 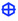 PS/APP&TPER-19/C15A-1 [25], PS/APP&TPER-18//C15A-2 [25], 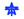 PS/APP&TPER-19/MWCNT-1 [25], PS/APP&TPER-18/MWCNT-2 [25], 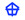 PS/APP&TPER-19/Fe_2_O_3_-1 [25], PS/APP&TPER-18/Fe_2_O_3_-2 [25], 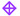 TPES/AlPi-30 [21]; and *FRI(t_i_&t_p_*) symbols are 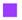 EP/DP-27.3 [14], Si-EP/DP-27.3 [14], 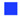 EP/DP-27.3/Mel-9.1 [14], Si-EP/DP-27.3/Mel-9.1 [14], 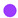 EP/BPA-BPP-9 [15], 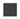 EP/GN-2 [16], 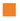 EP/Ni–Fe LDH-2 [16], 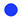 EP/NiFe-LDH&GN-2 [16], 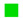 ABS/LIG-30 [17], 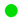 ABS/P-LIG-30 [17], 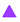 EVA/APP-10 [18], 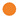 EVA/ATH-25 [19], 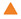 EVA/Boehm-25 [19], 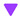 Cop-PA66&PA6/SiDOPO-10 [20], 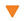 PA66/GF-35 [21], PA12/GF-30 [21], PEEK/GF-30 [23], 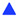 PA66/GF&RP-35 [21], 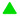 PBS/Cellulose-30 [22], 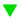 PBS/Bamboo-30 [22], 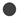 PEEK/CF-30 [23], 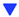 PP/BrFR&Sb_2_O_3_-20 [24], PP/BrFR&Sb_2_O_3_-25 [24], PS/BrFR&Sb_2_O_3_-12 [24], 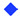 PS/BrFR&Sb_2_O_3_-10/C15A-2 [24], 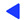 PS/BrFR&Sb_2_O_3_-10/MWCNT-2 [24], 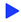 PS/APP&TPER-20 [25], 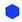 PS/APP&TPER-19/C15A-1 [25], PS/APP&TPER-18//C15A-2 [25], 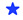 PS/APP&TPER-19/MWCNT-1 [25], PS/APP&TPER-18/MWCNT-2 [25], 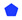 PS/APP&TPER-19/Fe_2_O_3_-1 [25], PS/APP&TPER-18/Fe_2_O_3_-2 [25], 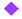 TPES/AlPi-30 [21].

Analyses performed in this work are indicative of the reliability of *FRI* for classifying polymer materials in terms of flame retardancy performance. Although the role of *t_p_* was unveiled in hybridized systems where “*Excellent*” flame retardancy performance was achieved, the category of flame retardancy performance remained unchanged. The variation of the *FI* as the measure of flammability versus *FRI* was also established (Figure 6). First, it can be recognized that the highest values of *FI* were obtained for samples containing halogenated FRs (symbols surrounded by blue dotted-line loops) with the action exclusively in the gas phase. However, significantly lower values of *FI* were obtained for some samples containing phosphorus or bio-based FRs (symbols surrounded by purple and green dotted-line loops, respectively), with an action principally in the condensed phase. This is basically expected, bearing in mind that barrier thermophysical effect is not effective in PCFC as a consequence of a very small sample size. On the other hand, the *FRI* values obtained for a given family of FRs would change significantly depending on the formula. This means that the inclusion of *t_p_* ended in a larger variance depending on the type of FRs. For instance, green symbols signifying bio-based FRs in Figure 6 take an ellipsoidal zone in Figure 6a, which remains almost unaffected by the inclusion of *t_p_*, either alone or in combination with *t_p_*. The green zone is not enlarged or even deformed in the corresponding figures of 6b and 6c, respectively. This supports the physics of the system, such that the bio-based FRs are burnt quickly in the vicinity of *Poor*-*Good* interface and could not principally be determinative. Contrary to this observation, the mauve ellipsoidal zone representative of phosphorus FRs was enlarged by the inclusion of *t_p_*. It is obvious that the type, amount, and hybridization of phosphorus FRs with other types expressively enlarged the *Good* zone. In this sense, the zone shadowed in mauve extended from Figure 6a (*FRI* (*t_i_*) was used, and varied in the interval 2.5–5.5) to Figure 6b (*FRI* (*t_p_*) was used, and varied in the interval 1.2–9.0) by replacement of *t_i_* by *t_p_*. Thus, it seems that the *FRI* allows a better discrimination of FR action compared with the *FI*. It should be noted that cone calorimetry and PCFC data are variable time to time even for a given sample, such that their standard deviations should be considered in interpretations.

**Figure 7:**
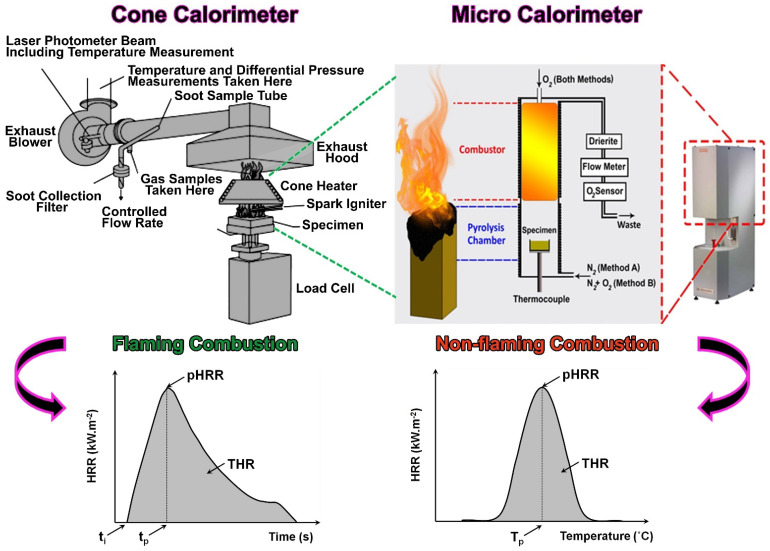
Cone calorimeter and PCFC combustion tests together with typical curves obtained from each technique, adapted from [33] with permission from Elsevier, 2022.

In conclusion, the reliability of the *FRI* in classifying polymer materials in terms of flame retardancy performance was assessed and reconfirmed. From a mechanistic view, cone calorimeter and PCFC tests together with typical curves obtained from these techniques were compared (Figure 7). Basically, both techniques make it possible to obtain the HRR curve. In PCFC, HRR is plotted as a function of the temperature, while in cone calorimeter it is a function of time. The pyrolysis and combustion are completely separated in PCFC, since these phenomena occur separately in two different chambers (Figure 7) [33]. Such a difference gives some points to cone calorimeter in view of the resemblance of a real fire scenario (flaming combustion). On the other hand, PCFC (non-flaming combustion) has been known for screening materials in view of the flammability. These facts lead to some difference in view of the performance measurement from two apparatus for a given material. In PCFC, a small quantity of material is pyrolyzed and the released gases are subsequently transformed to another chamber to experience a non-flaming combustion [33]. Therefore, the flame inhibition cannot principally be detected in PCFC. Moreover, due to the small size of the sample (2–4 mg), the barrier insulating effect does not exist [26]. In the cone calorimeter test, however, both phases (flame and condensed) exist simultaneously; therefore, all actions contribute to combustion (flame inhibition, barrier, and thermophysical effects) and can be detected [34]. All in all, *FRI* and *FI* can facilitate and unravel the efficiency of FRs added to polymers. They can also classify flame-retardant polymers into *Poor*, *Good*, and *Excellent* groups. Interestingly enough, the powerfulness and versatility of cone calorimeter over PCFC was reflected in the dominance of *FRI* over *FI*. Nevertheless, a generalization of outcomes of this survey would be possible only by collecting, analyzing, screening, and classifying plenty more data on both cone calorimeter and PCFC. In such systems, various kinds of FRs should individually or simultaneously be incorporated, varying their amounts to collect sufficient data for demonstration of the relationship between *FRI* and *FI*.

## Figures and Tables

**Table 1:** Cone calorimetry data taken from the literature to visualize the flame retardancy performance of polymer composites using Equations (2)–(4), i.e., *FRI* (*t_i_*), *FRI* (*t_p_*), and *FRI* (*t_i_*&*t_p_*); along with the values of the *FI* calculated using Equation (5) based on Pyrolysis Combustion Flow Calorimetry (PCFC) data available. The names of incorporated FRs are given in each case.

Polymer and Incorporated FR	FR (wt.%)	Heat Flux (kW·m^−2^)	*t*_i_ (s)	*t*_p_ (s)	pHRR (kW·m^−^²)	THR (MJ·m^−^²)	*FRI* (*t_i_*)	*FRI* (*t_p_*)	*FRI (t_i_*&*t_p_*)	T_p_ (°C)	pHRR (W·g^−1^)	THR (kJ·g^−1^)	*FI*	Refs.
Epoxy (EP)	0	35	38	77	2550	96	—	—	—	385	545	30	—	[14]
EP/6H-dibenz[c,e][1,2]oxaphosphorin,6-[(1-oxido-2,6,7-trioxa-1-phosphabicyclo[2.2.2]oct-4-yl)methoxy]-, 6-oxide (DP)	20.3	35	31	80	744	61	4.40	5.60	4.57	331	371	24	1.57	[14]
EP/DP/Melamine (Mel)	27.05	35	50	94	730	62	7.11	6.60	8.68	336	297	26	1.84	[14]
Silanized epoxy with 2 wt.% silica (Si-EP)	0	35	40	77	1964	79	—	—	—	388	448	28	—	[14]
Si-EP/DP	20.3	35	28	122	516	68	3.09	7.00	4.90	347	372	24	1.25	[14]
Si-EP/DP/Mel	27.05	35	42	102	909	57	3.14	3.96	4.16	340	270	26	1.56	[14]
EP	0	35	82	135	1148	88.4	—	—	—	395.9	709.6	32.8	—	[15]
EP/Bisphenol A bridged penta(anilino) cyclotriphosphazene (BPA-BPP)	9	35	72	150	457	78.4	2.48	3.14	2.76	352.8	433.8	26	1.83	[15]
EP	0	35	68	130	1730	113.1	—	—	—	401	449	24.4	—	[16]
EP/Graphene (GN)	2	35	86	155.5	980	65.1	3.87	3.66	4.64	398	285	20.3	1.87	[16]
EP/Ni–Fe layered double hydroxide (Ni–Fe LDH)	2	35	80	120.2	1070	58.9	3.65	2.87	3.37	427	240	17.9	2.71	[16]
EP/NiFe-LDH&GN	2	35	89	141.2	678	44.2	8.54	7.09	9.28	440	189	16.4	3.87	[16]
Acrylonitrile-butadiene-styrene (ABS)	0	35	80	157	482	72	—	—	—	340	602.6	37.7	—	[17]
ABS/Kraft lignin (LIG)	30	35	49	153	275	63	1.22	1.95	1.19	320	411.7	29.2	1.77	[17]
ABS/Phosphorylation of lignin (P-LIG)	30	35	49	167	202	58	1.81	3.15	1.93	340	411	27.2	2.03	[17]
Ethylene vinyl acetate copolymer (EVA)	0	35	65	175	1588	108	—	—	—	473	919	37.8	—	[18]
EVA/Ammoniumpolyphosphate (APP)	10	35	28	144	1030	93	0.77	1.47	0.63	466	758	31.6	1.42	[18]
EVA	0	50	39	180	1366	135	—	—	—	490	800	34.8	—	[19]
EVA/Aluminum trihydroxyde (ATH)	25	50	37	145	710	121	2.03	1.72	1.64	490	572	27	1.80	[19]
EVA/Precipitated boehmite (Boehm)	25	50	54	180	612	122	3.41	2.46	3.42	489	538	25.5	2.02	[19]
Polyamide 66/Polyamide 6 (90:10 wt.%) copolymer (Cop-PA66&PA6)	0	50	77	250	886	140.1	—	—	—	458	618	26.9	—	[20]
Cop-PA66&PA6/Organophosphorous alkoxysilane (SiDOPO)	10	50	62	186	597	104.8	1.59	1.47	1.18	443	468	25.2	1.36	[20]
PA66	0	50	51	149	1509	100	—	—	—	465	633	30	—	[21]
PA66/Glass fiber (GF)	35	50	43	92	582	80	2.73	2.00	1.68	455	354	18	2.91	[21]
PA66/GF/Red phosphorus (RP)	35	50	43	57	299	66	6.44	2.92	2.46	390	201	18	4.40	[21]
Polyamide 12 (PA12)	0	50	63	185	2205	164	—	—	—	475	937	35	—	[21]
PA12/GF	30	50	36	155	1992	153	0.67	0.99	0.56	477	762	31	1.39	[21]
Polybutylene succinate (PBS)	0	35	150	284	485	873	—	—	—	410	394	18.4	—	[22]
PBS/Cellulose	30	35	96	298	385	984	0.71	1.17	0.75	420	275	14.9	1.81	[22]
PBS/Bamboo	30	35	43	107	339	884	0.40	0.53	0.15	413	293	15.1	1.65	[22]
Poly(oxy-1,4-phenyleneoxy-1,4-phenylenecarbonyl-1,4-phenylene) (PEEK)	0	50	110	182	415.2	36.2	—	—	—	619	303	10.7	—	[23]
PEEK/Carbon fibre (CF)	30	50	156	279	146.7	26.9	5.40	5.83	8.28	621	195	7	2.38	[23]
PEEK/GF	30	50	115	278	120.5	23.3	5.59	8.17	8.54	623	233	7.2	1.94	[23]
Polypropylene (PP)	0	35	47	168	1573	140	—	—	—	486	1228	41	—	[24]
PP/Decabromodiphenyl oxide&Sb_2_O_3_ with 5:1 (BrFR&Sb_2_O_3_)	20	35	57	135	1445	84	2.20	1.45	1.76	470	374	33	3.94	[24]
PP/BrFR&Sb_2_O_3_	25	35	52	131	1177	61	3.39	2.39	2.64	459	318	30	4.98	[24]
Polystyrene (PS)	0	35	44	180	1166	101	—	—	—	441	1046	38	—	[24]
PS/BrFR&Sb_2_O_3_	12	35	55	128	591	46	5.41	3.08	3.85	410	598	33	1.87	[24]
PS/BrFR&Sb_2_O_3_/Cloisite15A (C15A)	12	35	35	115	442	43	4.92	3.95	3.14	432	408	33	2.89	[24]
PS/BrFR&Sb_2_O_3_/Multiwall carbon nanotubes (MWCNT)	12	35	34	141	340	43	6.22	6.30	4.87	429	341	31	3.65	[24]
Polystyrene (PS)	0	35	44	180	1166	101	—	—	—	441	1046	38	—	[25]
PS/Ammonium polyphosphate (APP)/Tripentaerythritol (TPER)	20	35	34	67	601	73	2.07	0.99	0.77	452	554	30	2.45	[25]
PS/APP/TPER/C15A	20	35	34	201	333	72	3.79	5.48	4.23	455	528	31	2.50	[25]
PS/APP/TPER/C15A	20	35	34	225	320	77	3.69	5.97	4.61	441	526	31	2.43	[25]
PS/APP/TPER/MWCNT	20	35	26	50	519	71	1.88	0.88	0.52	451	581	30	2.33	[25]
PS/APP/TPER/MWCNT	20	35	32	41	457	69	2.71	0.85	0.61	448	605	30	2.22	[25]
PS/APP/TPER/Nanoparticle Fe_2_O_3_ (Fe_2_O_3_)	20	35	28	78	456	74	2.22	1.51	0.96	451	581	31	2.25	[25]
PS/APP/TPER/Fe_2_O_3_	20	35	32	37	467	75	2.44	0.69	0.50	449	536	31	2.43	[25]
Styrene Ethylene Butylene Styrene&PP (TPES)	0	50	23	196	2346	215	—	—	—	447	565	43	—	[21]
TPES/AlPi	30	50	23	245	1048	160	3.00	3.76	3.76	440	462	37	1.39	[21]

**Table 2:** *Excellent* cases based on *FRI* analyzing cone calorimetry data on PP and EP as typical of thermoplastics and thermosets, respectively. Equations (2)–(4) are used to calculate *FRI* (*t_i_*), *FRI* (*t_p_*), and *FRI* (*t_i_*&*t_p_*), respectively. Unfortunately, the *FI* values could not be calculated because of a lack of data on PCFC. In most cases, hybridization of FRs was behind *Excellent* performance.

	Polymer and Incorporated FR	FR (wt.%)	Heat Flux (kW·m^−2^)	*t*_i_ (s)	*t*_p_ (s)	pHRR (kW·m^−^²)	THR (MJ·m^−^²)	*FRI* (*t_i_*)	*FRI* (*t_p_*)	*FRI* (*t_i_*&*t_p_*)	T_p_ (°C)	pHRR (W·g^−1^)	THR (kJ·g^−1^)	*FI*	Refs.
	Epoxy (EP)	0	35	52	90	1334.3	58.8	―	―	―	―	―	―	―	[28]
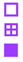	EP/Piperazine-modified ammonium polyphosphate (PAz-APP)	15	35	33	55	246.1	11.3	17.90	17.24	10.94	―	―	―	―	[28]
	EP	0	35	57	130	1730.27	114.16	―	―	―	―	―	―	―	[29]
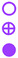	EP/Ammonium polyphosphate (APP)	15	35	63	95	397.89	35.49	15.46	10.22	11.29	―	―	―	―	[29]
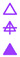	EP/Glycidyl methacrylate microencapsulated ammonium polyphosphate (GMA-APP)	15	35	68	160	283.09	44	18.91	19.51	23.28	―	―	―	―	[29]
	Epoxy (EP)	0	35	57	120	459	55.2	―	―	―	―	―	―	―	[30]
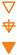	EP/Boric oxide (BO)	40	35	68	243	82	20.6	17.89	30.37	36.23	―	―	―	―	[30]
	EP	0	50	23	67	1910	61	―	―	―	―	―	―	―	[31]
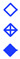	EP/Melamine coated ammonium polyphosphate/Talc (Mel-APP/Talc)	29.7	50	28	60	357	24	16.55	12.17	14/82	―	―	―	―	[31]
	Polypropylene (PP)	0	35	37	189	363	56	―	―	―	―	―	―	―	[32]
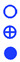	PP/Nitrogen –phosphorus contained intumescent flame retardant/Octadecyl trimethyl ammonium bromide modified montmorillonite (IFR/OTAB-MMT)	28	35	31	360	45	18	21.02	47.80	40.05	―	―	―	―	[32]
	PP	0	35	37	189	363	56	―	―	―	―	―	―	―	[32]
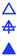	PP/Nitrogen–phosphorus contained intumescent flame retardant/Aminopropylisobutyl polyhedral oligosilsesquioxane (IFR/A-POSS)	28	35	32	375	55	16	19.97	45.83	39.63	―	―	―	―	[32]

## Data Availability

The data are available upon reasonable request from the corresponding author.
